# Hardness Perception Based on Dynamic Stiffness in Tapping

**DOI:** 10.3389/fpsyg.2018.02654

**Published:** 2019-01-04

**Authors:** Kosuke Higashi, Shogo Okamoto, Yoji Yamada, Hikaru Nagano, Masashi Konyo

**Affiliations:** ^1^Department of Mechanical Systems Engineering, Nagoya University, Nagoya, Japan; ^2^Graduate School of Information Sciences, Tohoku University, Sendai, Japan

**Keywords:** hardness perception, dynamic stiffness, frequency, vibration, principal component analysis

## Abstract

A human can judge the hardness of an object based on the damped natural vibration caused by tapping the surface of the object using a fingertip. In this study, we investigated the influence of the dynamic characteristics of vibrations on the hardness perceived by tapping. Subjectively reported hardness values were related to the dynamic stiffness of several objects. The dynamic stiffness, which characterizes the impulsive response of an object, was acquired across the 40–1,000 Hz frequency range for cuboids of 14 types of materials by administering a hammering test. We performed two psychophysical experiments—a ranking task and a magnitude-estimation tasks—wherein participants rated the perceived hardness of each block by tapping it with a finger. We found that the perceptual effect of dynamic stiffness depends on the frequency. Its effect displayed a peak around 300 Hz and decreased or disappeared at higher frequencies, at which human perceptual capabilities are limited. The acquired results help design hardness experienced by products.

## 1. Introduction

In daily life, a person may tap the surface of an object to judge its hardness when the object is rigid and cannot be deformed by pinching or pushing. Thus far, the principle of hardness perception by tapping has yet to be studied extensively. The general conclusion among previous studies seems to be that the dominant frequency of the transient vibrations yielded by tapping is a major cue for judging the hardness-related properties of objects (Okamura et al., 2001; Kuchenbecker et al., 2006; Ikeda and Hasegawa, 2009; Higashi et al., 2015, 2018a). Greater frequencies lead to greater perceived hardness. Other studies have revealed intriguing properties, although repeated testing and confirmation of these properties in various conditions is necessary before they can be widely accepted. For example, the presence of multiple frequency components in a single tapping event was reported to produce realistic stimuli (Kuchenbecker et al., 2006). The viscosity or damping properties of an object may also determine the hardness perceived by tapping as well as the stiffness of the object (Higashi et al., 2016b). Despite these previous investigations, few researchers have studied the vibrational frequency characteristics associated with the hardness perceived by tapping. In contrast, thus far, the contributions of force cue have been intensively studied. The maximum value and the rising steepness of the reaction force against an impulsive input force influence the hardness perceived by striking an object (LaMotte, 2000; Lawrence et al., 2000; Friedman et al., 2008; Han and Choi, 2010; Higashi et al., 2018b). Also, when discriminating deformable objects by using a fingertip, material elasticity and static stiffness, which is defined by the ratio of static pressing force to an object surface and its indentation depth, are known as major cues (Srinivasan and LaMotte, 1995; Bicchi et al., 2000; Friedman et al., 2008; Bergmann Tiest and Kappers, 2009; Scilingo et al., 2010; Kaim and Drewing, 2011). Especially, for objects as soft as or softer than human skin, the skin deformation cue plays more important role than kinesthetic one (Friedman et al., 2008; Bergmann Tiest and Kappers, 2009).

Another aspect that should be mentioned is the rapid growth of the usage of vibrotactile feedback functions in consumer products. Compared with softness or compliance (Srinivasan and LaMotte, 1995; Bicchi et al., 2000; Bergmann Tiest and Kappers, 2009; Kaim and Drewing, 2011; Metzger and Drewing, 2017; Hauser and Gerling, 2018), hardness perception by tapping has not been reported on extensively, potentially because of its limited commercial applications. However, vibrotactile methods of expressing object collisions were proven to be valuable (Okamura et al., 2001; Kuchenbecker et al., 2006; Cao et al., 2017; Culbertson and Kuchenbecker, 2017; Gongora et al., 2017; Hachisu and Kajimoto, 2017; Park and Choi, 2017) and are increasingly common in consumer products. Studying the mechanisms of hardness perception by tapping will be helpful for developing effective rendering techniques.

In previous studies in which force or vibrotactile displays have been used to present tapping events virtually based on transient vibrations, vibratory stimuli have tended to be simplified to damped vibrations with single frequency components. However, in reality, the response of an object to an impulsive tapping force includes a wide range of frequency components. Therefore, in the present study, we investigated the perceptual effects of frequency characteristics on hardness perception in a wide range from 40 to 1,000 Hz. To this end, we used the dynamic stiffness of a variety of objects. Dynamic stiffness is frequency-dependent and exhibits different values depending on the speed of object deformation. Furthermore, it expresses the unique frequency characteristics associated with the hardness of the object. We linked the dynamic stiffness and subjective hardness of objects using multivariate analysis and determined the impact of the dynamic stiffness in each of several frequency bands. The dynamic stiffness was investigated by performing a mechanical test using an impulse hammer, and the subjective hardness was determined by conducting two types of psychophysical tests, which were the ranking and magnitude estimation tasks, using human participants.

This study is an extended version of an earlier study conducted by the authors (Higashi et al., 2017), in which the connection between dynamic stiffness and subjective hardness was demonstrated. Nonetheless, they used a psychometrics that were converted based on a ranking task with strong assumptions of the distribution of answers. Furthermore, they did not shutout the information of weights and surface roughness of specimens used in the experiment. The weight pertains to the density of object and apparently influences the subjective hardness. The roughness perception is slightly correlated with the hardness perception in several reports in a manner that the rougher objects are felt the harder (Yoshida, 1968; Tamura et al., 2000; Picard et al., 2003; Soufflet et al., 2004; Sakamoto and Watanabe, 2017). In the present study, we conducted two types of psychophysical experiments (i.e., ranking task and magnitude estimation task) to calculate two types of psychometrics and investigated the effects of dynamic stiffness. Furthermore, the surfaces of specimens were finely finished, and the experimental protocol was designed such that participants would not know the weight of the specimen.

## 2. Materials and Methods

### 2.1. Ethical Statements

This study was approved by the internal review board of the School of Engineering, Nagoya University (#15-12).

### 2.2. Hardness Specimens

Fourteen types of specimens made of different materials were used in both the mechanical and subjective tests. Each specimen was a solid cuboid made of one of the 14 types of materials listed in Table 1. The cuboids were composed of plastic, wood, metal, rubber, and stone, which are popular materials in daily life. Each of the materials was sufficiently rigid such that it would not be deflected when its surface was pushed with a fingertip. This condition blocked participants from judging hardness based on the surface deformation. The surfaces of natural materials such as cork were finely polished such that their surface textures would not affect the material recognition.

**Table 1:** Materials and sizes of hardness specimens.

**Material**	**Size [mm]**
Acrylic resin	60 × 60 × 30
Polycarbonate resin	60 × 60 × 30
Nylon resin	60 × 60 × 30
Nitrile rubber	60 × 60 × 30
Urethane rubber (soft)	60 × 60 × 30
Urethane rubber (hard)	60 × 60 × 30
Wood	60 × 60 × 30
Wax	60 × 60 × 30
Stainless steel	60 × 60 × 30
Aluminum	60 × 60 × 30
Granite	100 × 100 × 30
Brick	100 × 100 × 60
Concrete	200 × 100 × 60
Cork	200 × 100 × 60

### 2.3. Hammering Test for Determining Dynamic Stiffness

#### 2.3.1. Dynamic Stiffness

Static stiffness is defined as the ratio of the force acting on an object to its surface displacement. In contrast, dynamic stiffness is the force per unit vibratory displacement and is defined in the frequency domain. The latter is frequency-dependent, whereas the former is not. For example, provided that the dynamic stiffness of an object is higher at 50 Hz than 100 Hz, the vibration amplitude is smaller at 50 Hz than at 100 Hz, even if the magnitude of excitation force is the same. Thus, the transient vibrations caused by tapping the object can be mathematically decoupled into an impulsive force input by tapping and the dynamic stiffness unique to that object.

The dynamic stiffness can be expressed in terms of the force *f*(*t*) and surface displacement *x*(*t*), given that we used an accelerometer to measure the acceleration *ẍ*(*t*) of the surface vibrations, as follows

(1) $$\begin{array}{l} H\left(\omega \right)=\frac{F\left[f\left(t\right)\right]}{F\left[x\left(t\right)\right]}=\frac{F\left[f\left(t\right)\right]}{F\left[ẍ\left(t\right)\right]/{\left(j\omega \right)}^{2}}, \end{array}$$

where F, ω, and *j* denote the Fourier transform, angular frequency, and imaginary unit, respectively. Both *ẍ*(*t*) and *f*(*t*) were measured and transformed into values in the frequency domain by performing Fourier transforms. Finally, we computed the absolute value of *H*(ω) for later analysis.

In our experiment, the dynamic stiffness was calculated based on the results of a hammering test. Considering the capabilities of the accelerometer that we used and human perception, we measured the stiffness across the 40–1,000 Hz frequency range.

#### 2.3.2. Experimental Setup

**Figure 1:**
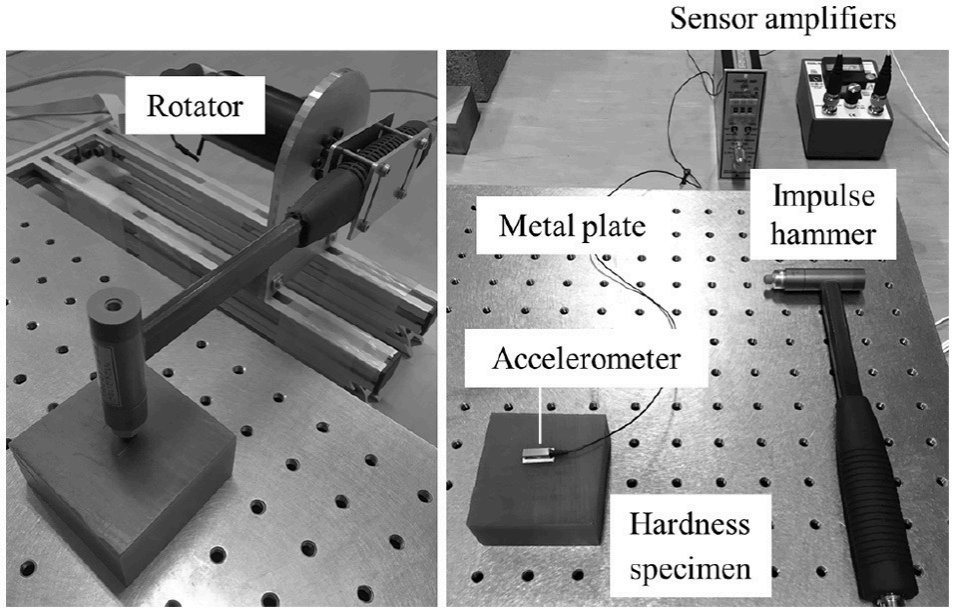
Measurement apparatus for the hammering test. The hammer was fixed to a rotator and released at a fixed angle.

Figure 1 shows the measurement apparatus used in the hammering test. An impulse hammer (GK-3100, Ono Sokki Co. Ltd., Japan) and amplifier (480M96A, Ono Sokki Co. Ltd.) intended for the load cell embedded in the hammer were employed. One end of the hammer was fixed to a rotator and released from rest at a certain height. A high-precision piezo accelerometer (2302B, Showa Sokki Co. Ltd., Japan, valid over 20 Hz) was fixed near the center of the surface that was to be struck by the hammer, and its output was acquired through an amplifier (4035-50, Showa Sokki Co. Ltd., Japan). The force and acceleration data were sampled using an oscilloscope at 10 kHz. Each specimen was fixed on a large metal plate (800 × 800 × 100 mm) for the hammering test.

**Figure 2:**
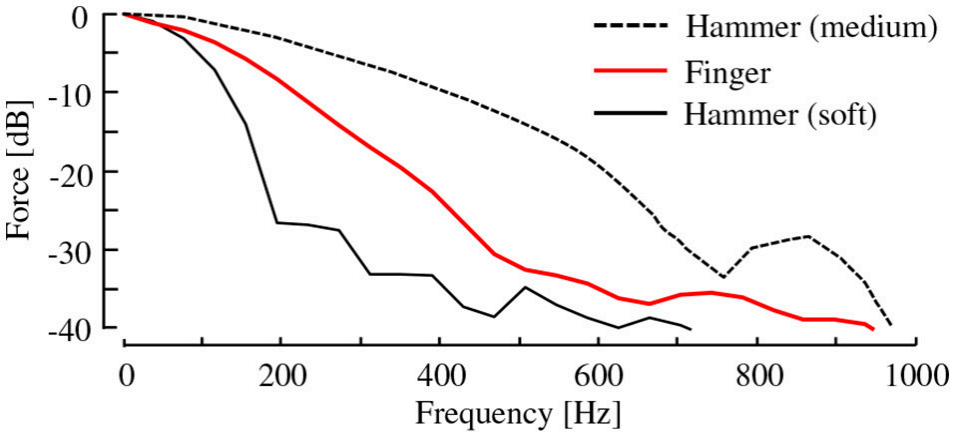
Impulse force spectra obtained using two hammer tips and fingertip.

Ideally, the frequency response of the hammer tip should be similar to that of a human fingertip. A few tips are commercially available for the impulse hammer we used. Among them, we chose the one whose frequency characteristics are the most similar to those of a human fingertip. Figure 2 shows the frequency spectra of the impulsive force when the two hammer tips and a fingertip were used. Unfortunately, the spectra of the fingertip lay between those of the two hammer tips. We selected the softer one, and it is not clear how the difference in spectra between the hammer tip and fingertip affects the latter analysis. It is intriguing to consider whether an actual fingertip can be used for the hammering test. However, in case using the actual fingertip, the correct placement of the load cell is a difficult challenge.

#### 2.3.3. Hammering Test Procedure

**Figure 3:**
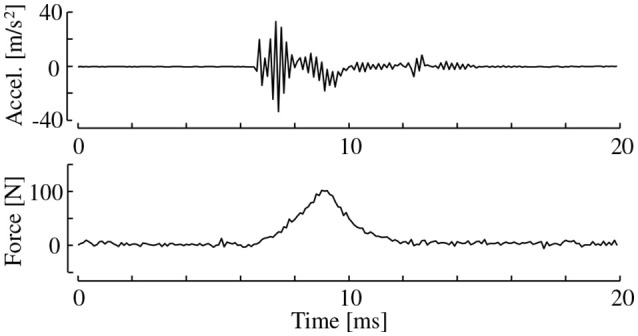
Example of surface acceleration and force data acquired by performing a hammering test (Hard urethane).

Each specimen was struck by the hammer at the center of its largest surface. Furthermore, each of the specimens was tested multiple times such that 10 valid data values were acquired. Invalid trials most commonly occurred due to protracted single strikes with double hammering or contact periods that were too long. These invalid trials were easily detected by checking the force records. Figure 3 shows an example of the force and acceleration acquired during a strike. The contact periods of valid trials were generally 2–3 ms.

#### 2.3.4. Dynamic Stiffness of Hardness Specimens

**Figure 4:**
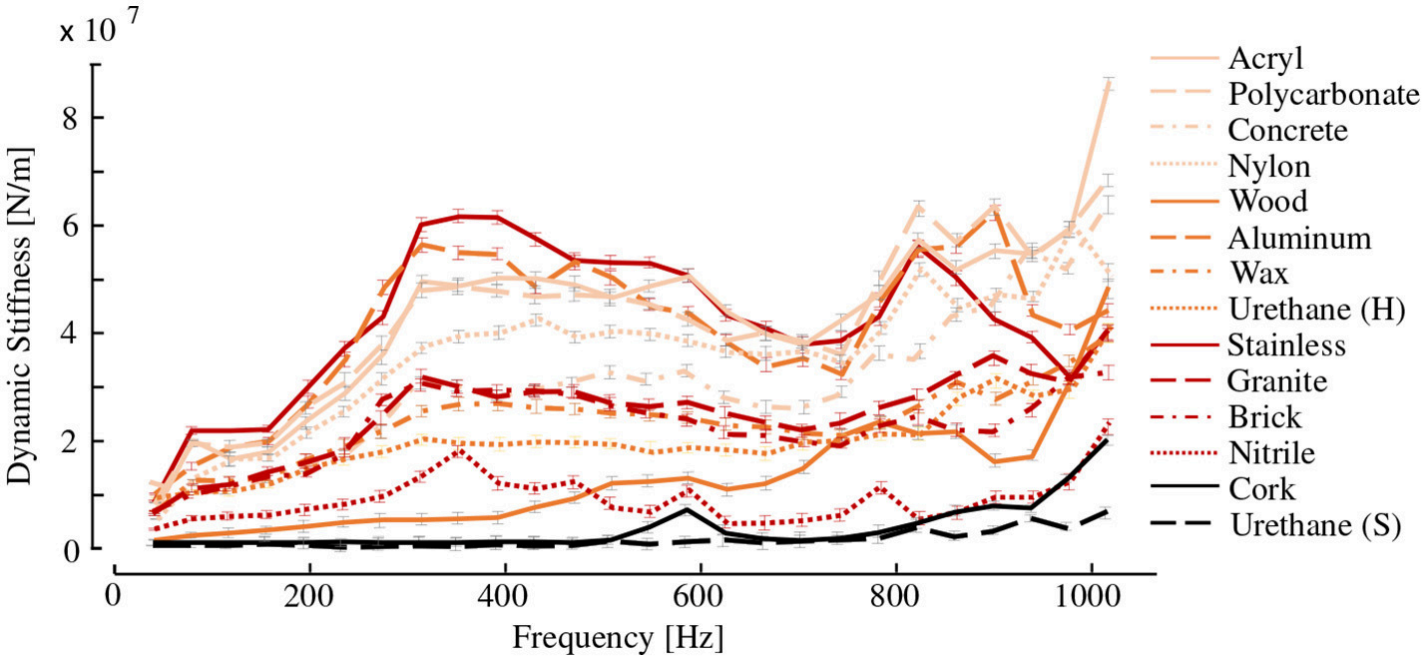
Dynamic stiffness of the material specimens. Mean and standard deviations acquired from 10 hammer strikes.

Figure 4 shows the dynamic stiffness computed for each of the 14 specimens. The dynamic stiffness values agree with our intuitive understanding of hardness. The specimens composed of materials that are typically perceived as hard, including aluminum, stainless steel, concrete, and polycarbonate, display relatively large stiffness values across the wide frequency range. In contrast, those composed of soft materials, including urethane rubber, nitrile rubber, cork, and wood, exhibit smaller stiffness values. The peak stiffness is different for each of the specimens, especially for the softer materials. However, the hard materials display similar dynamic stiffness values in the low frequency range.

### 2.4. Psychophysical Experiments for Subjective Ratings of Hardness Specimens

To investigate the effects of psychometrics, the subjective hardness of each specimen was rated in two types of psychophysical experiments, i.e., ranking and magnitude estimation tasks. The results of these two types of experiments were separately analyzed and compared.

#### 2.4.1. Participants

The participants were eight male university students who agreed to participate in the study and provided informed consent. All of the participants were in their 20s, were right-handed, and declared no haptic deficit. Two tasks were performed by all the participants. Half of the participants performed the ranking task first, whereas the other half began with the magnitude estimation task.

#### 2.4.2. Experiment 1: Ranking Task

##### 2.4.2.1. Procedures

Fourteen types of hardness specimens were randomly placed on a desk. The participants compared and ranked them by tapping their surfaces. The number of taps was not specified, and a participant could assign the same ranks to multiple specimens (i.e., multiple specimens could be perceived to have the same hardness).

Each participant was instructed to tap the center of the largest area of each specimen using the index finger of his writing hand. To avoid judgment based on the thermal properties, neither placing a finger on the specimen nor pushing was allowed. Nonetheless, the thermal cues could not be fully controlled in the present study. It is not clear how heat transfer during a short contact period might affect material recognition. Furthermore, to avoid judgment based on the density or heaviness of the specimens, the experimenter lifted and moved the specimens on the desk following requests from each participant. During the experiment, the participants wore headphones that played a pink noise to shut out the sounds of tapping. Since the participants wore sunglasses with opaque film, judgment based on the appearance of the specimens was impossible. Note that the specimen cuboids could barely be seen through the glasses. A total of two sets of trials were performed by each participant with a break of a few minutes between sets. Most of the participants were able to finish the ranking task within 10 min.

##### 2.4.2.2. Data analysis

For better application of the multivariate analyses described below, we converted the rank of each specimen assigned by the individual participants into an interval scale using a normalized ranking method (Harter, 1961). This method assumes that random samples from a normal distribution with an average and standard deviation of zero and one, respectively, are ranked in the order of observed values. The method converts the rank of a sample to its expected value on this assumption. The range of normalized ranks depends on the number of samples, in this case ±1.70 based on 14 samples. The normalized ranks of each specimen were averaged across the participants and were used in the subsequent analysis.

#### 2.4.3. Experiment 2: Magnitude Estimation Task

##### 2.4.3.1. Procedures

The participants evaluated the subjective hardness of the specimens using the magnitude estimation method without a modulus. Specifically, each participant evaluated the subjective hardness of each specimen as a numerical value by tapping its surface. They could use positive real numbers, including fractions and decimals. The participants followed the same instructions regarding how to tap the specimens as in Experiment 1. The visual and auditory cues were blocked by headphones playing pink noise and by sunglasses with opaque film. The specimens were presented one-by-one in random order. Fourteen specimens were evaluated in a single set, and a total of three sets were performed by each participant. Most of the participants were able to finish each set within 10 min.

##### 2.4.3.2. Data analysis

No significant differences in reported hardness scores were found among the three trials. The data from all trials were used for the subsequent analysis. The scores were normalized across participants to bring all data onto a common scale and reduce the influence of the participants' arbitrary choice of scale. The hardness scores were then geometrically averaged among the participants for each specimen. These geometric averages were used in the subsequent analysis.

### 2.5. Relation Between Dynamic Stiffness and Perceived Hardness Based on Multivariate Analysis

#### 2.5.1. Correlation Between Single Component of Dynamic Stiffness and Subjective Hardness

**Figure 5:**
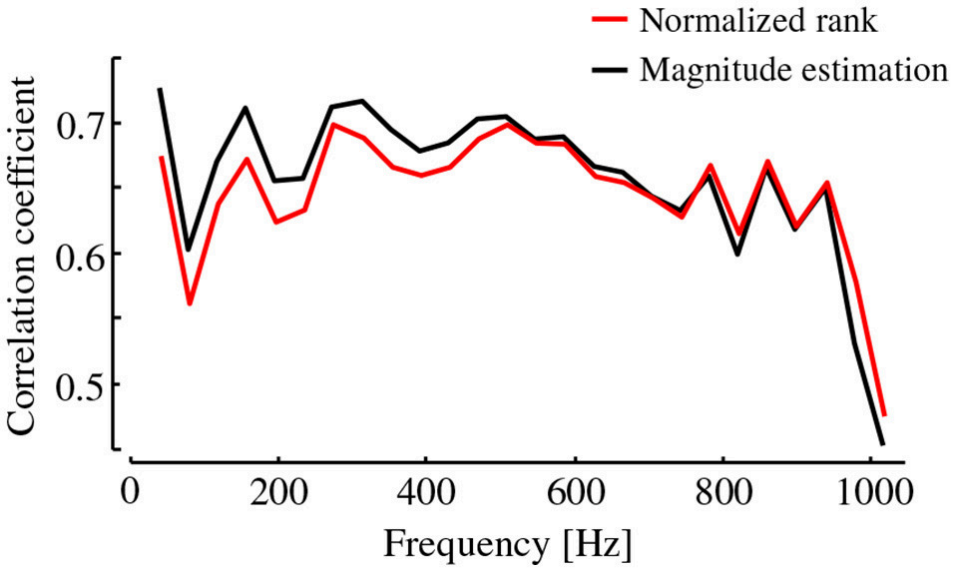
Correlation coefficients between the dynamic stiffness at each frequency and two types of subjective hardness. The effect of dynamic stiffness on subjective hardness depends on the frequency.

Although our main interest lies in the quantitative connections between the magnitudes of dynamic stiffness and hardness perception, herein, their simple correlation coefficients are checked. Figure 5 shows correlation coefficients between the subjective hardness and the dynamic stiffness values of specimens for each frequency component. The correlation coefficients calculated from the two types of subjective scores produced similar profiles. The correlation coefficients varied in the range of about 0.5–0.7 depending on the frequency. Such frequency dependency suggested that the perceptual weightings of frequencies are not equal across the whole frequency range. It should be noted that these simple correlation coefficients do not directly indicate the relationships between the dynamic stiffness and hardness perception when the dynamic stiffness values at different frequencies are correlated with each other. Especially, the correlations varied in a zigzag manner with peaks at 40 and 160 Hz with a valley at the center of these two peaks at 80 Hz. There is no rationale for explaining this zigzag profile in terms of the characteristics of human's vibrotactile perception.

#### 2.5.2. Prediction Model and Analysis Strategy

Since the frequency of the damped vibrations produced by tapping influences the perceived hardness (Okamura et al., 2001; Kuchenbecker et al., 2006; Ikeda and Hasegawa, 2009; Higashi et al., 2016a) and the perception of a vibrotactile stimulus significantly depends on its frequency (Bolanowski et al., 1988), the effect of dynamic stiffness on hardness perception should also depend on the frequency of the stimulus. We related the dynamic stiffness values $s_{i}\in {\mathbb{R}}^{m\times 1}$ of *m* representative frequencies for specimen *i* to the subjective hardness *h*_*i*_ using

(2) $$\begin{array}{lll} h_{i} & = & s_{i}^{T}w+c\\ = & \overset{m}{\underset{j}{\sum }}s_{ij}w_{j}+c, \end{array}$$

where *s*_*ij*_, *w*_*j*_, and *c* are the stiffness at the *j*th representative frequency for specimen *i*, its perceptual weight, and a constant that represents the intercept of the hardness scores, respectively. The first and lowest representative frequency was 40 Hz, while the last and highest one was 1,015 Hz. The number of representative frequencies was 26, and they were separated by equal intervals of 39 Hz: *m* = 26. The weight ***w*** that best relates *h*_*i*_ to ***s***_*i*_ suggests how the dynamic stiffness influences the subjective hardness. Hence, the objective of this analysis was to determine such ***w*** values.

For *n* dynamic stiffness samples, the simultaneous equations can be expressed as

(3) $$\begin{array}{l} h=Sw+c, \end{array}$$

where $S={\left[s_{1},s_{2},\ldots ,s_{n}\right]}^{T}$ and $h={\left[h_{1},h_{2},\ldots ,h_{n}\right]}^{T}$ are the dynamic stiffness matrix and hardness score vector, respectively. Matrix ***S*** ∈ ℝ^*n*×*m*^ was composed of the stiffness values for *m* representative frequencies for *n* types of specimens: *n* = 14. Vector ***h*** ∈ ℝ^*n*×1^ comprised the subjective hardness scores of the 14 specimens.

Because of the issue of the degree of freedom (i.e., *n* < *m*), (3) cannot be solved directly by using the generalized inverse matrix of ***S***. Even if a number of samples are tested and *n* > *m*, the collinearity property of the dynamic stiffness values is raised as a problem. Collinearity makes the computation of the generalized inverse matrix unstable because of the rank deficit caused by high correlation coefficients among explanatory variables, which are the dynamic stiffness values. To avoid these issues, we employed an approach described in the following sections.

#### 2.5.3. Multiple Regression Analysis Using Principal Components of Dynamic Stiffness

To avoid the abovementioned computational problem, we used the principal components of the observed dynamic stiffness. With a varimax rotation applied, ***S*** was decomposed into the component scores *A* ∈ ℝ^*n*×*m*′^ of all trials and the principal component vectors *B* ∈ ℝ^*m′*×*m*^ by

(4) $$\begin{array}{l} S\;\sim \;AB, \end{array}$$

where *m*′ < *m*. The number of principal components was determined to be three such that the cumulative contribution ratio reached 95%: *m*′ = 3.

**Figure 6:**
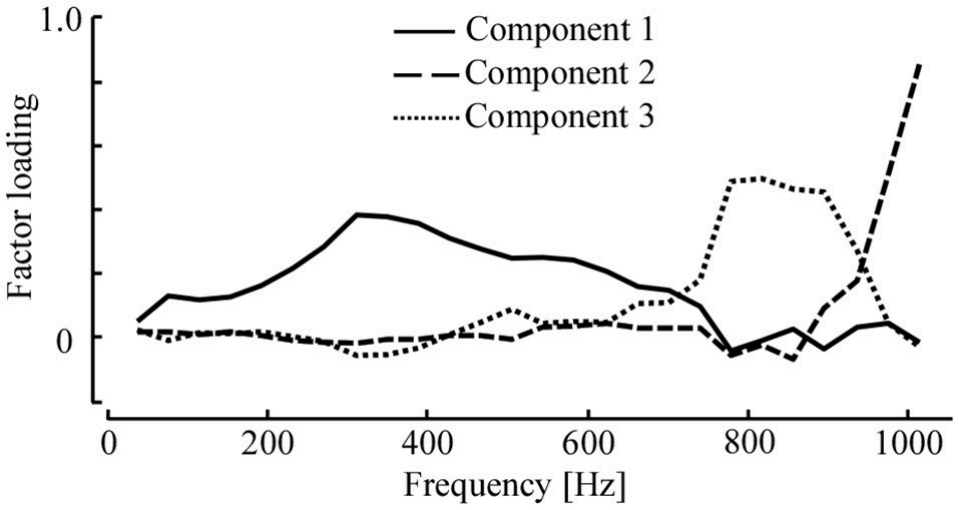
Loading vectors of three principal components for the dynamic stiffness of 14 specimens. The loadings are scaled by their eigenvalues. The first component includes stiffness below 800 Hz with the peak around 300 Hz. The second component includes the stiffness at 800–900 Hz, while the third component weights the stiffness above 900 Hz.

Hence, by using vectors, (4) can be rewritten as

(5) $$\begin{array}{l} S\;\sim \;\left[a_{1}\;a_{2}\;a_{3}\right]{\left[b_{1}\;b_{2}\;b_{3}\right]}^{T}, \end{array}$$

where $a_{k}\in {\mathbb{R}}^{n\times 1}$ is the score vector of principal component $b_{k}\in {\mathbb{R}}^{m\times 1}$. The three components, ***b***_1_, ***b***_2_, and ***b***_3_, are shown in Figure 6. The first component, ***b***_1_, was largely loaded by the stiffness in the middle frequency range of approximately 200–500 Hz. In contrast, the second component, ***b***_2_, was loaded at high frequencies of nearly 900–1,000 Hz. The third component, ***b***_3_, lay between ***b***_1_ and ***b***_2_, and was loaded by the stiffness at 700–900 Hz.

We then performed multiple regression analysis with the principal component scores and subjective hardness scores being explanatory and objective variables, respectively. For this analysis, the subjective hardness ***h*** was modeled as a product of ***a*** vectors and regression coefficients $z={\left[z_{1}\;z_{2}\;z_{3}\right]}^{T}\in {\mathbb{R}}^{m^{\prime }\times 1}$ and was determined based on

(6) $$\begin{array}{l} h\;\sim \left[a_{1}\;a_{2}\;a_{3}\right]{\left[z_{1}\;z_{2}\;z_{3}\right]}^{T}+c. \end{array}$$

The use of principal components allowed us to avoid overfitting because the number of explanatory variables was merely *m*′ = 3. Furthermore, this method does not include the problem of collinearity because the explanatory variables are mutually independent.

Using the stepwise method, we selected ***a***_*i*_ vectors that significantly affected ***h***. As a result, ***a***_1_ [*t*_0_(138) = 11.6, *p* < 0.001] and *a*_3_ [*t*_0_(138) = 2.43, *p* = 0.016], which were the scores for the first and third principal components ***b***_1_ and ***b***_3_, respectively, were found to influence the hardness scores statistically. Hence, using the scores for these two components, the regression equation can be rewritten as

(7) $$\begin{array}{l} h\;\sim \left[a_{1}\;a_{3}\right]{\left[z_{1}\;z_{3}\right]}^{T}+c. \end{array}$$

Based on (5), (7) is identical to

(8) $$\begin{array}{l} h\;\sim \;S{\left[b_{1}\;b_{3}\right]}^{+}{\left[z_{1}\;z_{3}\right]}^{T}+c, \end{array}$$

where ^+^ indicates the generalized inverse matrix. Here, $w\;\sim \;{\left[b_{1}\;b_{3}\right]}^{+}{\left[z_{1}\;z_{3}\right]}^{T}$ is regarded as the perceptual weights against the stiffness for *m* representative frequencies.

## 3. Results

### 3.1. Subjective Hardness Scores

**Figure 7:**
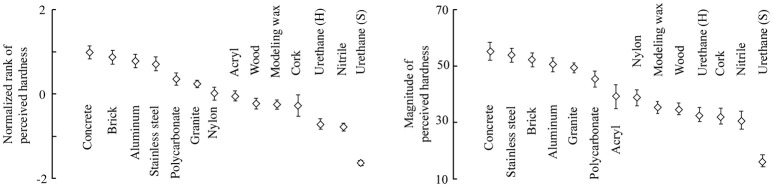
Perceived hardness values of the specimens, including the means and standard errors of the hardness scores among the participants. **(Left)** Values based on the ranking task. **(Right)** Values based on the magnitude estimation task.

Figure 7 **(left)** shows the mean and standard error of the normalized ranking scores for each specimen. The standard error for each specimen was small compared to the differences in their means, which indicates that the individual differences in the scores were small. The stone and metal cuboids were ranked the highest, followed by plastic, wood, and rubber. The hardness ranks were largely consistent with our intuitive understanding of hardness. That is, ostensibly hard specimens were ranked as such, and vice versa. In the subsequent analysis, the mean scores were used as representative subjective hardness values. Figure 7 **(right)** shows the geometric means and standard errors of the reported hardness scores for all of the specimens. The stone and metal cuboids were scored the highest, followed by the plastic, wood, and rubber ones. The hardness ranks of the specimens were largely consistent between the two types of psychometrics.

### 3.2. Weightings of the Dynamic Stiffness

**Figure 8:**
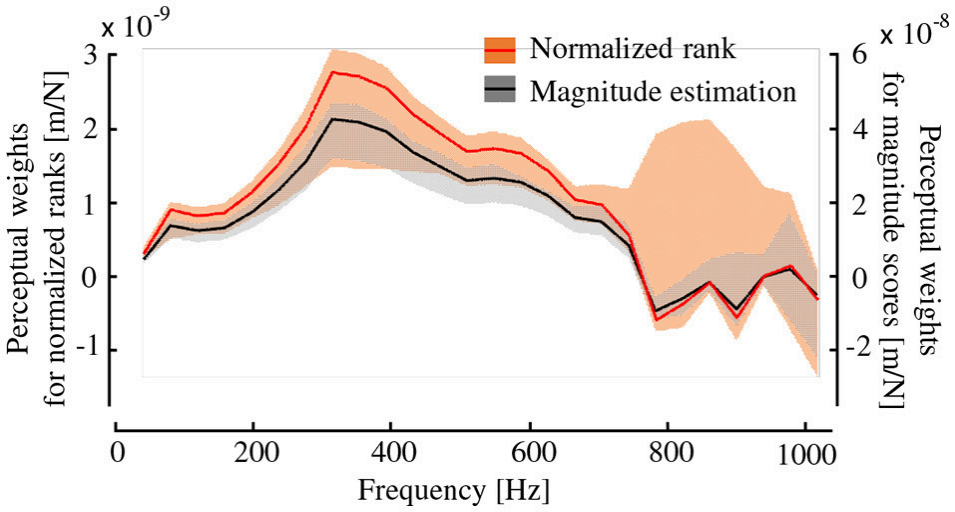
Frequency characteristics of the weight function for the subjective hardness and dynamic stiffness. Weights for the two types of subjective hardness are shown with shades being the maximum and minimum range. The perceptual weights of dynamic stiffness values at approximately 300 Hz are the greatest whereas those above 800 Hz are nearly zero.

Figure 8 shows the perceptual weights ***w*** of the dynamic stiffness values from 40 to 1,015 Hz. This figure includes the results obtained using the two types of psychometrics. A constant value *c* was −0.75 and 28.8 for the ranking and magnitude estimation tasks, respectively. The shaded regions represent the maximum and minimum values of variation of ***w*** found in the validation in which one of the 14 types of hardness specimens was removed from the analysis to determine whether the results were sensitive to or robust against the sample data. The type of psychometrics hardly influenced the results, and peaks were observable around 300 Hz for both types of psychometrics. The ***w*** values calculated from the two types of subjective scores produced similar profiles.

**Figure 9:**
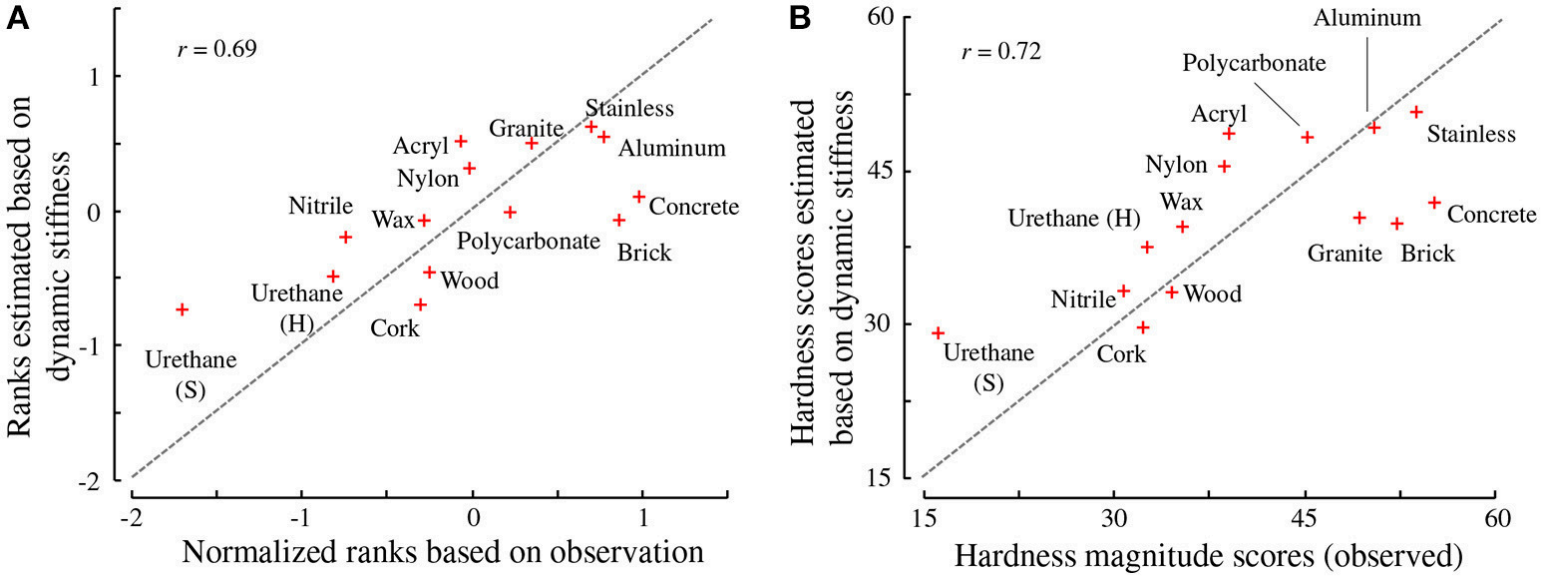
Correspondence between the observed (reported) and estimated hardness values. **(A)** Ranking task estimates. **(B)** Magnitude estimation method estimates. The dotted lines indicate the equality of the observed and estimated values.

We conducted a leave-one-out cross validation method where each model estimated the hardness score of a specimen that was not involved to establish the model. Figures 9A,B compare the hardness scores reported by the participants and those estimated based on the dynamic stiffness of the specimens. The correlation coefficients between the observed and estimated values were determined to be 0.69, when the normalized ranked scores were used and 0.72, when the values from the magnitude estimation method were used.

When performing regression analysis, the regression coefficients *z*_1_ and *z*_3_ in (8) were 6.0E-9 and 2.6E-9, respectively, for the normalized rank scores, and they were 1.0E-7 and 2.4E-8, respectively, for the scores of the magnitude estimation task. Hence, the first principal component influenced the subjective score more than the third component did. Nonetheless, both components were good predictors. The correlation coefficients between the subjective hardness scores and each of the two components were high. The first component exhibited a correlation coefficient of 0.69 for the normalized ranks and 0.72 for the scores of the magnitude estimation task. The third component exhibited a correlation coefficient of 0.67 for both types of scores. Both principal components were equally correlated with the hardness scores. Although these results apparently suggest the contribution of the third component, which mainly includes the dynamic stiffness at 800–900 Hz, as discussed later in section 4, the resultant perceptual weights shown in Figure 8 does not support the effects of the dynamic stiffness above 800 Hz.

The hardness values of certain specimens were not accurately predicted. These specimens were the urethane, concrete, and brick cuboids, which were reported to be the softest or hardest among all the specimens in the psychophysical experiments. This behavior is typical for a linear prediction model that linearly approximates limited parts of nonlinear phenomena. Our linear model, (3), is applicable to a limited set of moderately hard specimens. It would be intriguing to search for a nonlinear estimation model that suits a wide range of specimens; however, the present experiments were not designed for such a purpose.

## 4. Discussion

Figures 9A,B demonstrate that the dynamic stiffness of an object is an effective predictor of the hardness perceived by tapping. In other words, hardness perception is likely to be based on the relationship between the force exerted on the object surface and its vibrations. The perceptual weight was positive or zero for the most frequency range, which indicates that greater stiffness led to greater perceived hardness. For the tapping of the object, the perceived hardness is positively correlated with the static stiffness defined by the quasi-static relationship between the force and deflection (Higashi et al., 2016b). The results of our study indicate that this correlation also holds for dynamic stiffness.

The weight values varied across the frequency range, suggesting that the perceptual effect of dynamic stiffness depends on the frequency. This dependence may be due to human vibrotactile characteristics, which are also frequency-dependent.

Human vibrotactile sensitivity is the highest around 250 Hz and deteriorates above and below this frequency (Bolanowski et al., 1988). At higher frequencies near 1 kHz, the perception of vibrotactile stimuli is difficult. Such sensitivity largely agrees with the weights derived from the principal component analysis. The weights were the greatest around 300 Hz, the range in which vibrotactile perception is the most sensitive. Above approximately 800 Hz, the range in which vibrotactile perception is unlikely to operate, the weights were nearly zero, suggesting that stiffness does not affect hardness perception in this frequency range.

As described in the introduction, higher frequency leads to the greater hardness perception, according to previous studies where the frequency used for vibrotactile stimuli was at most 300 Hz (Okamura et al., 2001; Ikeda and Hasegawa, 2009; Higashi et al., 2015, 2018a; Culbertson and Kuchenbecker, 2017). As shown in Figure 7, the weight monotonically increases up to approximately 300 Hz, which means that (at least beneath this frequency) a higher frequency is more influential on hardness perception. Hence, the derived weights are consistent with the findings in previous studies.

It should be noted that the generality of the findings remains to be studied. In this study, we used cuboids made of several types of materials. The dimensions of some were different from the others. Both the material and structure of an object influence the softness perceived by pinching or pushing it (Bergmann Tiest and Kappers, 2009). Similarly, dynamic stiffness of an object depends on both the material of which it is composed and its dimensions, because an object's dimensions influence its vibration modes. Hence, we did not exclusively investigate the differences among the materials. To investigate the effects of dimensions, however, we need a set of specimens made of the same materials but of different sizes. Such specimens would help us test the generality of this study.

## 5. Conclusion

Humans judge the hardness of objects using vibratory cues generated by tapping. However, little is known about how humans leverage such transient vibrotactile cues. To search for the relationships between the mechanical characteristics of objects and the hardness perceived by tapping in a wide frequency range, we investigated the relationship between the dynamic stiffness values and subjective hardness scores of a variety of cuboids. We found that the dynamic stiffness across the 40–800 Hz frequency range can be used effectively to estimate the perceived hardness with the peak weightings around 300 Hz. The frequency-dependent contributions to perception can be reasonably interpreted based on the vibrotactile characteristics of humans. These findings help understand the mechanism of hardness perception in tapping objects.

## Author Contributions

KH and SO equally contributed to the work including the design, preparation, and execution of the experiments, analysis, and manuscript preparation. YY, HN, and MK equally contributed to advising the study and proof-reading of the manuscript.

### Conflict of Interest Statement

The authors declare that the research was conducted in the absence of any commercial or financial relationships that could be construed as a potential conflict of interest.
